# Patterns of Prescription Medicine, Illicit Drugs, and Alcohol Misuse among High-Risk Population: A Factor Analysis to Delineate Profiles of Polydrug Users

**DOI:** 10.3390/healthcare10040710

**Published:** 2022-04-11

**Authors:** Alessio Gili, Massimo Lancia, Isabella Mercurio, Mauro Bacci, Alessia Nicoletti, Chiara Pelliccia, Cristiana Gambelunghe

**Affiliations:** 1 Hygiene and Public Health Section, Department of Medicine and Surgery, University of Perugia, Piazza Lucio Severi, 06132 Perugia, Italy; alessio.gili@gmail.com; 2 Forensic Medicine, Forensic Science and Sports Medicine Section, Department of Medicine and Surgery, University of Perugia, Piazza Lucio Severi, 06132 Perugia, Italy; massimo.lancia@unipg.it (M.L.); isabmerc@gmail.com (I.M.); mauro.bacci@unipg.it (M.B.); nicolettialessia8@gmail.com (A.N.); chiarapelliccia02@gmail.com (C.P.)

**Keywords:** polydrug use, prescription drug misuse, alcohol abuse, illicit drugs, factor analysis, hair analysis

## Abstract

Polydrug use is a serious health and social problem worldwide. Treatment remains a challenge because it requires planning based on estimates of the nature and extent of drug consumption and the characteristics of the population in need. To this end, 103 subjects, who voluntarily asked to begin rehabilitation treatment, were monitored through hair analysis to investigate the nature and extent of their polydrug use. A factor analysis was carried out to delineate polydrug user profiles based on the following variables: age, sex, type of illicit drug use, type of prescription drug misuse, and amount of alcohol consumption. Twenty-three percent of subjects tested positive to more than one illicit drug (mainly cocaine), 44% to unprescribed drugs (mainly benzodiazepines), and 66% were hard drinkers. The profiles of drug users outlined included “single drug cocaine user”, and “single drug opiate user”. Moreover, a particularly problematic profile of cocaine users, common between genders and age groups, who combine high levels of alcohol and unprescribed benzodiazepines and opiates, emerged (“hard polydrug abusers”). From a treatment policy perspective, these findings support the importance of preventive analysis before rehabilitation treatment begins in order to identify different patterns of drug abusers to implement personalized multidisciplinary measures.

## 1. Introduction

Drug use patterns have changed dramatically over recent years. In this context, polydrug use is the rule rather than the exception worldwide, causing a serious public health problem with high toxicity, increased risk of accidents or injuries, overdose, and death as some of the main consequences [1]. It concerns the consumption of multiple drugs by one person, including the use of these drugs on separate occasions (sequential use) or at the same time (concurrent/simultaneous) [1]. The main effect sought by combining multiple drugs is a new or synergistic effect and an increase in the overall psychoactive experience. Other reasons relate to the reduction of the effects of other drugs, such as benzodiazepines (BZDs), the reduction of the insomnia caused by the use of stimulants, the relief of withdrawal symptoms from other drugs, the replacement of a drug which is lower in price or one which is more available, or simply for legality or fashion purposes [2,3]. Examples include cocaine replacing ecstasy, methadone substituting heroin, and gamma-butyrolactone (GBL) replacing gamma-hydroxybutyric acid (GHB) after GHB came under drug law control [2]. The use of more than one illicit drug, combined with alcohol and prescription drugs, has significantly increased and has consistently been associated with worse treatment outcomes, including poor treatment retention, higher rates of relapse, and a three-fold higher mortality rate compared to mono-substance use [3,4]. Moreover, the effects of some psychoactive substances can escalate the risk of using other substances. For example, alcohol intoxication can impair judgement about the amount of opioids (OPI) consumed or increase the risk of reduced tolerance after leaving treatment or prison [1]. Regarding the pattern of mixing stimulant drugs with alcohol, a report indicates that in Europe about 70% of adolescents first use cocaine (COC) under the effects of alcohol [5]. The combined use of both substances is particularly dangerous because of the increased risk of cardiovascular events, dysfunctional behavior (mostly related to disinhibition and impaired judgment), and premorbid vulnerability to psychiatric disorders such as depression, anxiety, and personality disorders (mainly antisocial and borderline) [6,7].

Another matter of increasing concern in Europe is the non-medical use of prescription drugs, particularly in light of the sharp rise in the number of deaths from prescription OPI analgesics in the United States [8,9]. The term “misuse of prescription drugs” or “non-medical use of prescription drugs” refers to the use of psychoactive drugs for self-medication, recreational or stimulant purposes, with or without a medical prescription, outside the official therapeutic indications and at doses often higher than recommended, frequently in the context of polydrug intake [10]. According to the 2015 National Survey on Drug Use and Health in the United States, prescription psychotherapeutic drugs (pain relievers, tranquilizers, stimulants, and sedatives) ranked second only to cannabis as the most prevalent illicit drug use category across all age groups [11]. Similar figures have been reported in European countries [11]. Prescription drugs are generally procured through family, acquaintances, legitimate prescriptions, at foreign destinations, or through internet purchases [12]. Moreover, the web, in addition to offering a wide range of products, provides numerous indications and tutorials on how to prepare and administer these drugs. Although these drugs are mainly taken orally, other routes, such as injection, smoking, or inhalation, have also been reported [12,13]. Among prescription drugs, sedatives and hypnotics are commonly misused. These include barbiturates; BZDs; BZD-like drugs such as z-hypnotics, OPI, and OPI- substitute medications that are used for pain relief; and stimulants to treat attention deficit and hyperactivity disorder [8]. Other medications, such as antidepressants (e.g., bupropion and venlafaxine), anti-Parkinson drugs, cough and cold medicines, stimulants such as methylphenidate, and second-generation antipsychotics (e.g., quetiapine and olanzapine), are also misused [13,14]. Recently, worldwide growing cases of the misuse of Pregabalin (PGB), an anticonvulsant and anxiolytic medication used to treat epilepsy and neuropathic pain, were also reported [15,16,17,18].

Polydrug use, including the misuse of prescription drugs, is common among adolescents and young adults, and in vulnerable subjects, such as those with a history of drug abuse [8]. The most common combinations of drugs recorded among drug treatment clients in Europe are cannabis (as the primary drug) consumed with alcohol and COC, OPI (as the primary drug) consumed with cannabis and COC, COC (as the primary drug) consumed with cannabis and alcohol, and non-COC stimulants (as the primary drug) consumed with alcohol and cannabis [1].

Based on what has been argued, it is clear that rapid changes in drug scenarios present a challenge for pharmacy, psychiatry, public health, and drug control policies [10]. Therefore, it is of fundamental importance to investigate and monitor the extent and nature of polydrug abuse for developing appropriate interventions to address the diverse needs for treatment and care of polydrug users. Depending on the specifics of a case, a multidisciplinary approach can be applied, including pharmacological (detoxification, opioid agonist, and antagonist maintenance), and/or psychosocial intervention (counselling, cognitive behavioral therapy, and social support), focusing on the process of rehabilitation, recovery, and social reintegration [19].

For this purpose, an at-risk population of 103 subjects with substance use disorders (SUDs), including drug and/or alcohol disorders, who sought to begin treatment at the Public Health Service for Drug Dependence, were monitored for alcohol and illicit drug use and misuse of prescription drugs through hair analysis, which can assess the extent of the abuse.

## 2. Materials and Methods

### 2.1. Subjects and Sample Collection

Laboratory procedures were conducted in accordance with the Helsinki Declaration of 1975 (revised 1983) and approved by the Bioethics Review Board of the University of Perugia (Protocol 2012-006R). All participants provided informed consent. The cohort consisted of 103 subjects (aged 18–59 years; 56 males and 47 females) from urban areas of central Italy, who had SUDs and had voluntarily sought treatment at the Public Health Service for Drug Dependence during 2019. The age distribution between the young and the adults was uniform since 48 subjects (46.60%) were below 30 years, and 55 subjects (53.40%) were above 30 years (Table S1).

A hair sample (approximately 3 cm in length, 50 mg) was collected from an area close to the scalp near the posterior vertex and submitted for a general screening for illicit drugs, prescription drugs, and alcohol abuse by determination of ethyl glucuronide (EtG).

All the subjects submitted their respective lists of prescription medications.

### 2.2. Reagents and Chemicals

Analytical grade solvents, reagents, analytical drug standards, and deuterated analogs were purchased from Merck (Milan, Italy).

### 2.3. Preparation and Analysis of the Hair Samples

All the hair samples were prepared, extracted, and derivatized according to previously described methods [17,20]. Analytical determination was carried out on a triple quadrupole (GC/MS-MS) 7000C GC/MS system (Agilent Technologies, Palo Alto, CA, USA) operated under the electron ionization (EI) mode and fitted with a 7890 B gas chromatograph (Agilent Technologies, Palo Alto, CA, USA). This system was equipped with an HP-5MS (Agilent Technologies, Palo Alto, CA, USA) capillary column (length 30 m, inner diameter 0.25 mm, and film thickness 0.25 mm), operated with helium at a flow rate of 1 mL/min and temperature programming of 80 °C for 1 min ramped at 8 °C/min to 300 °C and held for 2 min. The samples (1 µL) were injected into a split–spitless injector at 250 °C in the spitless mode (1 min). The ion source and AUX temperatures were 250 °C and 280 °C, respectively. MS acquisition for unknown substances was performed in the full-scan mode in the range of 41–500 amu. Analytes were identified by matching experimental full-scan spectra against the NIST spectral library, the most powerful database for screening unknown substances. PGB and EtG determination was carried out in the GC/MS-MS system operated in multiple reaction monitoring (MRM) mode, precursor ion: m/z 172 and m/z 261, respectively [17,20].

### 2.4. Statistical Analysis

Descriptive statistics were performed using frequencies, percentages, frequency tables for categorical variables, and mean ± standard deviation (SD) for quantitative variables.

Tetrachoric correlations were evaluated for binary variables that described the use of each substance. Tetrachoric correlations assumed a latent bivariate normal distribution (X1, X2) for each pair of variables (v1, v2), with a threshold model for the manifest variables, vi = 1, if and only if Xi > 0. The means and variances of the latent variables were not identified, but the correlation r of X1 and X2 can be estimated from the joint distribution of v1 and v2 and is called the tetrachoric correlation. The pairwise correlation matrix was returned as r (Rho) and was used to perform a factor analysis of the binary variables. Factor analysis/correlation (principal factors method) with orthogonal varimax rotation and Kaiser normalization [21] was performed to explain the covariance or the correlation between the substances used. A scree plot visual tool was implemented to determine the optimal number of factors (Figure S1). The plot suggests that we retained three factors, both because of the shape of the scree plot and because of the Kaiser criterion that suggests retaining factors with eigenvalues significantly larger than 1 [22]. In accordance with guidelines suggested by Taherdoost et al. [23], all substances and characteristics with abs (load) < 4 were excluded as they did not consider weak correlation levels.

Statistical analyses were performed using STATA 16.1 (Stata Statistical Software: Release 16, College Station, TX, USA).

## 3. Results

The hair analysis investigated alcohol and illicit drug use and misuse of prescription drugs over three months in 2019 in 103 subjects (56 males and 47 females; age range 18–59 years), before they began a voluntary rehabilitation treatment at the Public Health Service for Drug Dependence of Umbria, a region of central Italy. As there were 439 new admissions in Umbria in 2019 [24], our study effectively analyzed 23.2% of the new cases. Hair was the selected modality of analysis, as it is a strong, stable tissue that is less affected by adulterants or short-term abstinence and has an advantage over traditional matrices (e.g., blood or urine), since long-term drug use history can be traced over a period of weeks to months, depending on the length of the hair collected [20]. Considering that the average hair growth is 1 cm per month, a hair sample 3 cm in length allowed us to examine a retrospective time window of approximately three months to determine which drugs were abused, and the level of alcohol consumed during the latest period before admission to the treatment rehabilitation service [20]. Moreover, hair sampling is simple, rapid, minimally invasive, and supports the identification and quantitation of multiple analyses per sample [20]. GC/MS general screening identified at least one illicit drug in the hair of all the examined subjects, and more than one illicit drug was found in 24 cases (23.3%). These were mainly COC (48 cases), similar figures of heroin (34 cases) and cannabis (33 cases), and MDMA (12 cases). The main combinations of illicit drugs were COC with OPI, COC with cannabis, and OPI with cannabis. Unprescribed drugs were detected in nearly half the cases (44), with BZDs (19), OPI (13), and PGB (8) being the most common drugs. Moreover, there were three cases of combined use of BZDs and OPI, and one case of combined use of BZDs and PGB. Alcohol use was monitored by measuring the variations of EtG in the hair, a useful biomarker for detecting high-risk drinking behavior. EtG is a minor ethanol metabolite present in several body fluids and tissues, including the hair [20]. Based on internationally adopted cut-off concentrations, abstinence from alcohol can be verified (EtG in hair <5 pg/mg), and chronic excessive drinking, with a consumption of 60 g or more of ethanol per day, can be detected (>30 pg/mg) [25,26].

High levels of alcohol use were detected in the hair of more than half of the subjects (68, 66.02%), and 43 of these 68 subjects (63.2 %) indicated misuse of prescription drugs.

A factor analysis was carried out to delineate the possible profiles of polydrug users based on the following variables: age, sex, type of illicit drug use, type of prescription drug misuse, and EtG values measured in hair.

**Figure 1:**
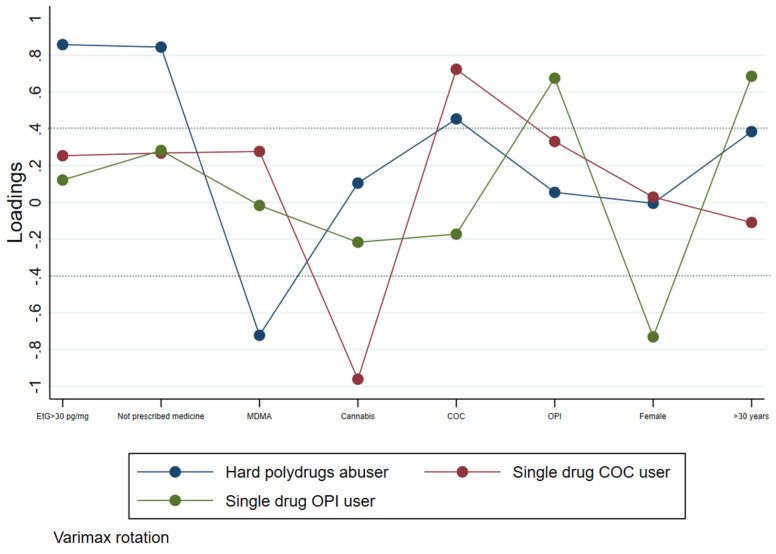
Profiles of drug users obtained by factors analysis.

Results from the analysis allowed us to outline three drug user profiles in the cohort examined, as shown in Figure 1 and Table 1.

We defined the first profile as the “hard polydrug abusers”, characterized by subjects in whom the primary drug of abuse was COC combined with the consumption of high levels of alcohol, and the misuse of prescription drugs, mainly BZDs (diazepam, alprazolam, lorazepam, flunitrazepam, and zolpidem), and OPI (codeine, fentanyl, and tramadol). Among these subjects, two cases of co-intake of COC and PGB were found. This profile was found in all age groups and genders. The other two profiles outlined were not related to combined polydrug use and alcohol drinking. In particular, we defined the second one as “single drug COC user”, characterized by cocaine abusers who do not consume prescription drugs and are moderate drinkers (<20 g ethanol/day). This profile belongs to all age groups and genders. In this group, a significant inverse correlation with cannabis use was observed.

Finally, the third profile was defined as “single drug OPI user”, characterized by opioids abusers. This profile was not related to the co-intake of high amounts of alcohol (>60 g ethanol/day) or to the misuse of prescription drugs, and it was primarily associated with male subjects over 30 years old.

According to the models developed, cannabis and MDMA users were less likely to take other drugs in combination with alcohol.

## 4. Discussion

Over the past decade, illicit drug use has been monitored through a combination of population- and substance-specific approaches. Conversely, the monitoring of the use of multiple substances and analyses of the overlaps between different populations of drug users have been limited. However, this is of particular importance in the context of public policies as the rising prevalence of multiple drug use (e.g., alcohol, cannabis, and COC) has resulted in additional populations of drug users with a growing range of available substances that allow them to try new combinations of drugs.

The data obtained in the present study show that, in a group of 103 subjects undergoing a rehabilitation process, more than half consume more than one drug in addition to the primary substance for which they had requested treatment, in accordance with the report [27] of the European Monitoring Center for Drugs and Drug Addiction (EMCDDA). Overall, 23% of the subjects consumed more than one illicit drug, and 66% were addicted to high levels of alcohol drinking.

A large number of subjects misused prescription drugs (42.7%), confirming the fact that the use of these drugs for non-therapeutic purposes is an emerging and increasingly problematic issue.

On this point, information from the European Drug Emergencies Network (Euro-DEN Plus), which monitors drug-related presentations in sentinel hospitals in a number of European countries, shows that approximately one-fifth of the presentations involve the non-medical use of prescription or over-the-counter medicines, most commonly OPI and BZDs [8]. Individuals suffering from mood swings, anxiety, physical and/or mental health problems, tension reduction/relaxation/euphoria, or drug withdrawal symptoms may crave the effects of such medications [8,28]. Results from our study agree with other studies that investigated polydrug use by hair analysis in different population groups. A Danish study [29] found concurrent use of BZDs and morphine, codeine, amphetamine, cannabis, cocaine, and ethanol among methadone-related fatalities. In Italy, a recent study [30] conducted among drivers through hair analysis showed that 12.15% were polydrug users, with cannabis and cocaine being the most common combination. Many studies have confirmed that cannabis is the most commonly used illicit drug in Europe [31]. The lowest proportion of polydrug users was found among primary cannabis users, similar to the heroin user profile in the examined cohort. They were not related to the problematic consumption of alcohol and prescription drugs.

Many indicators point to a potential increase in the problems related to COC use in Europe, such as 213 tons of drugs being seized in 2019; as such, the number of people undergoing treatment for the first time has increased over the past five years [31].

The spread of cocaine abuse is underlined in our study by the identification of two profiles in which cocaine was the main drug of abuse: the “hard polydrug abusers”, which combine the consumption of cocaine, alcohol, and prescription drugs (mainly OPI and BZDs), and the “single drug COC user”, which defines subjects who predominantly use cocaine.

Unlike other recreational drugs, which are often associated with specific sub-cultures and settings, COC has a more universal profile, and its various forms and routes of administration allow its effects to be tailored [32]. Users often seek energy, endurance, a feeling of ease and friendliness during social contacts, and sexual stimulation, finding it easier to control the duration and intensity of the effects of COC than those of the other drugs [32].

Help from social health services is generally required only after the user has developed severe health and social problems, which appear with frequent or heavy COC use [32]. Psychosocial interventions (e.g., cognitive behavioral therapy, motivational interviewing, and brief interventions), contingency management, management of psychiatric disorders, and symptomatic pharmacological treatment are the most commonly applied interventions when treating users seeking support for COC dependence in Europe [32]. Since there is also a significant prevalence of psychiatric comorbidity among polydrug users, an integrated intervention with mental health services is often required [32].

COC may cause significantly acute and chronic toxicity, which can manifest as neuro-psychiatric and sympathomimetic/stimulant effects, such as increased heart rate, increased blood pressure, hyperthermia, acute renal failure, and seizures [33]. Mental health risks associated with COC include dependence, agitation, anxiety, restlessness, insomnia, paranoia, auditory hallucinations, and chest pain, as well as depressive symptoms in the long run [32,33].

Subjects who consume COC with high quantities of alcohol make up the majority of those who seek treatment for COC-related problems in Europe [29], a finding reflected by our cohort.

If consumed with alcohol, COC combines in the liver to form cocaethylene, a psychoactive homologue with a plasma half-life longer than that of COC [34].

Cocaethylene temporarily enhances the high associated with both COC and alcohol but produces a major toxic effect, particularly in the cardiovascular system [34]. Several indices of neuro-psychological performance were found to be negatively affected by the concurrent intake of COC and alcohol compared to either drug administered alone [35]. From the user’s perspective, concurrent COC and alcohol use enhances and prolongs euphoria associated with COC, and ameliorates the unwanted feelings associated with the diminishing effects of stimulant drugs such as anxiety, agitation, and paranoia [34]. Moreover, users often experience that by drinking a high quantity of alcohol they can maintain a relatively high level of perceived sobriety [32].

The profile of “hard polydrug abusers” outlined in the cohort was also related to the combined use of prescription drugs, mainly BZDs and OPI. The misuse of prescription drugs has become a serious and widespread public health problem, which is often combined with the use of illicit drugs. Reasons for mixing COC with central nervous system depressants such as BZDs include decreasing the effects of COC, self-medicating for sleeplessness and psychiatric disorders, and attempting to counteract the dysphoric side effects of stimulant drugs [32]. Even in the case of “speedball” that involves injecting a combination of an OPI with a stimulant (e.g., COC or methamphetamine), the sought-after effect is generally to intensify the desired effects, and to suppress the unpleasant effects of both the drugs [32]. However, mixing stimulants and depressants such as BZDs and OPI increases the side effects of both the substances, aggravates symptoms of co-occurring mental or substance use disorders, increases the risk of addiction and/or dependence on either substance, and greatly increases the risk of fatal overdose [32].

## 5. Conclusions

The co-use of multiple substances can complicate the delivery of drug treatment, resulting in worse outcomes. Recognizing both polydrug use by patients seeking assistance and the severe health consequences of drug interactions is critically important for treatment, as they are sometimes overlooked. The toxicological investigation of the hair analysis of the cohort allowed for the recognition of a large number of subjects using multiple drugs, including misuse of prescription drugs. Moreover, it was possible to identify a particularly problematic profile of COC users, common between genders and age groups, who combined high levels of alcohol and unprescribed BZDs and OPI. The profiles outlined through toxicological monitoring before beginning treatment may contribute to the development of a suitable therapeutic response. Moreover, it is advisable that integrated, multidisciplinary programs based on delineating polydrug use patterns become routine during the entire rehabilitation program. This can be of great utility for the specific management of the health and social needs of well-delineated drug user profiles. Furthermore, the provision of real-time monitoring would allow rapid corrective actions to be taken in therapeutic treatment.

## Figures and Tables

**Table 1:** Correlation between factors and variables (Rotated factor loadings and unique variances).

Variables	Hard Polydrugs Abuser	Single Drug COC User	Single Drug OPI User	Uniqueness
**EtG > 30 pg/mg**	0.86			0.18
**Not prescribed medicine**	0.85			0.13
**MDMA**	−0.72			0.40
**Cannabis**		−0.97		0.02
**COC**	0.46	0.72		0.24
**OPI**			0.68	0.43
**Female**			−0.73	0.46
**>30 years**			0.69	0.37
Blanks represent abs (loading) < 4				

## Data Availability

The data presented in this study are available on request from the corresponding author. The data are not publicly available due to privacy restrictions.

## Supplementary Materials

The following supporting information can be downloaded at: https://www.mdpi.com/article/10.3390/healthcare10040710/s1, Table S1: Main characteristics of the cohort; Figure S1: Scree plot of the factor eigenvalues (horizontal green line was plotted at the mean of the eigenvalues).
