# Supplementary figures and images for: Patterns of Prescription Medicine, Illicit Drugs, and Alcohol Misuse among High-Risk Population: A Factor Analysis to Delineate Profiles of Polydrug Users
Source: Healthcare (Basel). 2022 Apr 11;10(4):710. doi: 10.3390/healthcare10040710 (PMC9031601; doi:10.3390/healthcare10040710)

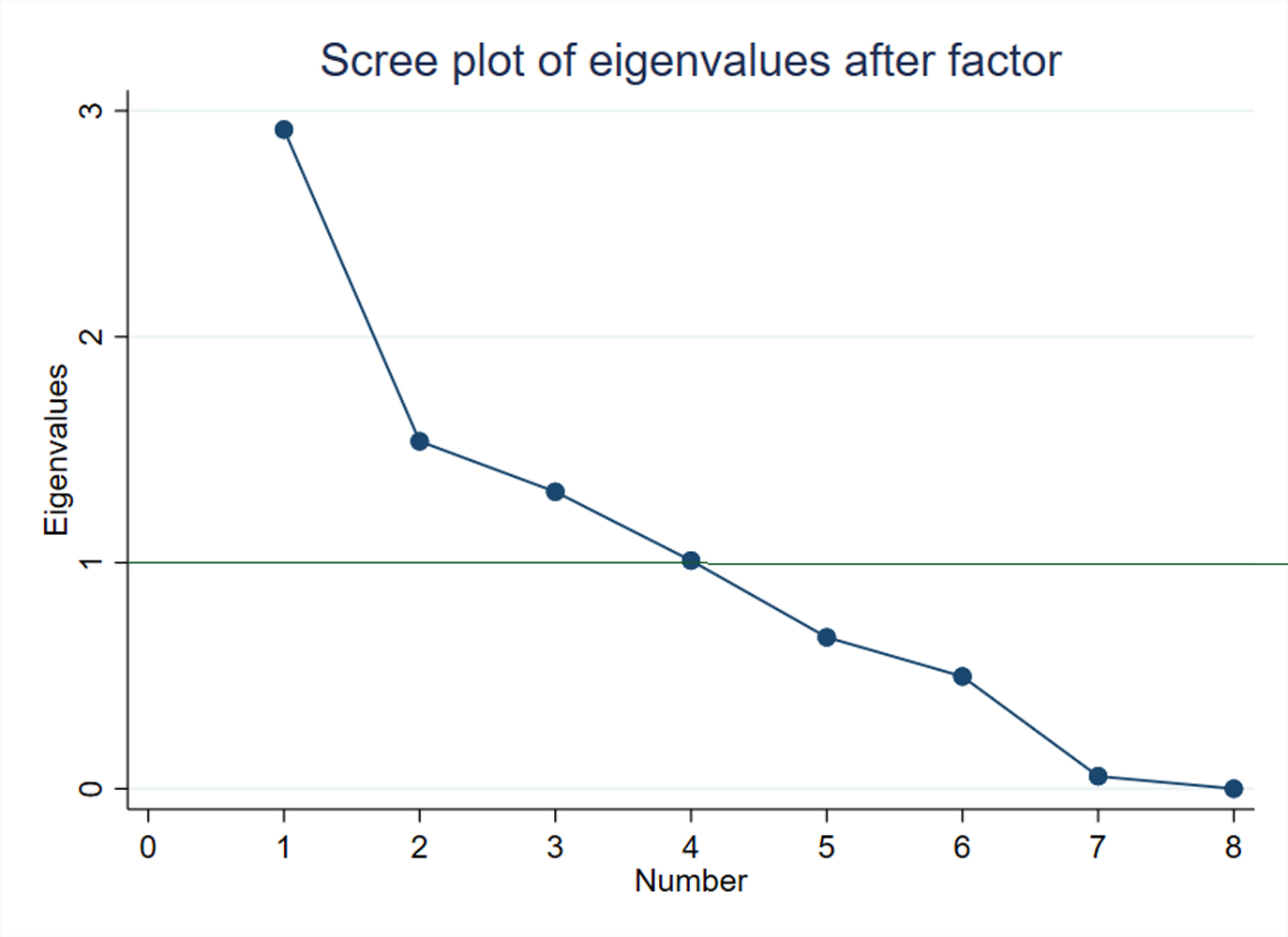

Supplement: Supplementary file 1 [file healthcare-10-00710-s001.zip › Figure S1.jpg]
